# Clinical Advances in Child Neurology

**DOI:** 10.3390/jcm15114398

**Published:** 2026-06-05

**Authors:** Claire-Marie Rangon, Debopam Samanta

**Affiliations:** 1 SMART-VNS Platform (Structured Multidisciplinary Platform to Advance Research on Therapy of Vagus Nerve Stimulation), Raymond Poincaré Hospital, AP-HP GHU Paris Saclay, 92380 Garches, France; clairemarie.rangon@aphp.fr; 2 Department of Pediatrics, University of Arkansas Medical Sciences, 1 Children’s Way, Little Rock, AR 72202, USA

When this Special Issue was launched, its initial aim was to gather innovative nonpharmacological therapeutic approaches for children with neurodevelopmental disorders. As submissions developed, however, the scope broadened to reflect the wider theme of Clinical Advances in Child Neurology. The final collection spans prognostic markers, measurement tools, novel biomarkers, pharmacological innovation, and mechanistic insights across diverse pediatric neurological conditions. Together, these papers offer a timely view of where child neurology stands—and where it must go next.

Any discussion of neurodevelopmental outcome must begin in the perinatal period. Keçeci and colleagues remind us that hypoxic–ischemic encephalopathy (HIE) remains a major cause of neonatal mortality and long-term neurodevelopmental impairment, despite the widespread use of therapeutic hypothermia [1,2]. Their retrospective analysis identifies early clinical and biochemical markers—including higher lactate levels, lower 5 min Apgar scores, and renal dysfunction—that may support more refined risk stratification and individualized follow-up [1]. This contribution anchors the Special Issue by emphasizing a central reality: the burden of neurodevelopmental disability is often shaped before a child ever reaches a neurology clinic or rehabilitation program.

The need for reliable, early, and accessible prognostic tools recurs throughout this collection. Datta’s review of interictal epileptiform discharges illustrates that even familiar biomarkers require careful interpretation [3]. Electroencephalography (EEG) remains central to the diagnosis and management of pediatric epilepsy, yet interictal discharges vary in topography, morphology, frequency, timing, and developmental significance [4]. Their prognostic meaning is therefore not uniform. This reinforces the need for nuanced, longitudinal, and multimodal assessment rather than reliance on isolated diagnostic signals [5].

The heterogeneity of pediatric neurology is further reflected in the report by Romeo et al. on mild encephalitis/encephalopathy with reversible splenial (MERS) lesion [6]. In contrast to conditions associated with severe and permanent impairment, MERS demonstrates that dramatic neuroimaging abnormalities may occur in a disorder with largely favorable outcomes [7]. In their cohort of 19 children, seizures, headaches, and drowsiness were common; infections, particularly viral agents such as rotavirus, were frequent; and hyponatremia was observed in many patients [6]. Importantly, nearly all the patients recovered fully [6]. MERS therefore sits at the intersection of infectious, metabolic, and neurological medicine, reminding clinicians that reversibility is itself an important neurological phenotype in children.

Neuroplasticity provides another unifying theme [8]. The developing brain’s capacity to adapt and reorganize is central to prognosis and intervention. Llamas-Ramos and colleagues contribute to this theme through their use of functional near-infrared spectroscopy (fNIRS) in infants, measuring bilateral oxyhemoglobin changes before, during, and after tactile interventions including massage and reflex locomotion therapy [9]. Their work highlights fNIRS as a promising, noninvasive tool for capturing early brain responses without the radiation exposure, sedation requirements, or cost barriers of many conventional neuroimaging approaches. Placed alongside the HIE study, this raises an important question: could longitudinal fNIRS help identify early prognostic markers in high-risk infants [10]?

Appling et al. extend the biomarker theme into attention-deficit/hyperactivity disorder (ADHD) [11]. In a small crossover pilot study, 17 participants completed creativity-related tasks on and off prescribed stimulant medication, with a pre-task electrocardiogram (EKG) used to derive heart rate variability measures [11]. The prespecified marker, baseline pNN50, did not predict stimulant-related improvement in divergent thinking or convergent problem-solving. Although negative, this finding is important [11]. It argues against premature use of resting pNN50 as a biomarker for stimulant-related cognitive or creative response, reduces publication bias, and helps constrain mechanistic hypotheses about autonomic versus dopaminergic contributions [11]. It also underscores the broader need for simple, noninvasive, physiologically meaningful predictors of treatment response [12,13,14].

Finally, the review of Lennox–Gastaut syndrome (LGS) addresses one of the most treatment-resistant developmental and epileptic encephalopathies [15]. Despite multiple approved antiseizure medications, optimal seizure control remains difficult [16]. The review evaluates emerging evidence for agents including perampanel, brivaracetam, cenobamate, stiripentol, and ganaxolone, emphasizing both the promise and uncertainty of newer pharmacological options in LGS management [17].

Read individually, each paper contributes to its own subspecialty. Read together, they construct a broader argument. First, pediatric neurology urgently needs better prognostic tools—from neonatal biomarkers in HIE, to EEG interpretation in epilepsy, to autonomic measures in ADHD. Second, measurement innovation is increasingly central to progress, with fNIRS, EEG, HRV, and other tools offering new ways to observe brain function and treatment response. Third, early action matters: the window for meaningful intervention is often narrow, particularly in infancy and early childhood. Fourth, therapeutic progress requires not only new treatments, but a clearer understanding of why treatments work, in whom, and under what biological conditions.

This Special Issue is therefore a contribution, not a conclusion. Its papers reflect a field moving toward earlier diagnosis, more precise prognostication, better physiological measurement, and more individualized treatment. We are grateful to the contributing authors, reviewers, and the editorial team at the Journal of Clinical Medicine for making this collection possible. The children who stand to benefit from the next generation of pediatric neuroscience deserve continued and accelerating effort.
